# Clinical Role of the Noninvasive Abdominal Fetal ECG in the Detection and Monitoring of Fetal Tachycardia

**DOI:** 10.1161/CIRCEP.124.013556

**Published:** 2025-09-11

**Authors:** Sian Chivers, Nicolò Pini, Shayan Chowdhury, Ludovica Cicci, Trisha Vigneswaran, Vita Zidere, Sophie Maxwell, Grace Moriarty, William Regan, Eric Rosenthal, David F.A. Lloyd, Thomas G. Day, Owen I. Miller, Gurleen K. Sharland, Barrie Hayes-Gill, Stephen Niederer, William P. Fifer, Catherine Williamson, John M. Simpson

**Affiliations:** 1 Department of Congenital Heart Disease, Evelina London Children’s Hospital, United Kingdom (S. Chivers, T.V., V.Z., S.M., G.M., W.R., E.R., D.F.A.L., T.G.D., O.I.M., G.K.S., J.M.S.).; 2 Department of Women’s and Children’s Health (S. Chivers, W.P.F., J.M.S.), King’s College London, United Kingdom.; 3 School of Biomedical Engineering and Imaging Sciences (D.F.A.L., T.G.D.), King’s College London, United Kingdom.; 4 Department of Psychiatry, Columbia University Irving Medical Center, New York, NY (N.P., S. Chowdhury, W.P.F.).; 5 Division of Developmental Neuroscience, New York State Psychiatric Institute (N.P., S. Chowdhury).; 6 Department of Biomedical Engineering (L.C., S.N.), Imperial College London, United Kingdom.; 7 Institute of Reproductive and Developmental Biology (C.W.), Imperial College London, United Kingdom.; 8 Faculty of Engineering, University of Nottingham, United Kingdom (B.H.-G.).

**Keywords:** atrial flutter, diabetes, gestational, fetus, heart rate, fetal, tachycardia, supraventricular

## Abstract

**BACKGROUND:**

Fetal tachycardias can cause adverse fetal outcomes including ventricular dysfunction, hydrops, and fetal demise. Postnatally, ECG is the gold standard, but, in fetal practice, echocardiography is used most frequently to diagnose and monitor fetal arrhythmias. Noninvasive extraction of the fetal ECG (fECG) may provide additional information about the electrophysiological mechanism and monitoring of intermittent arrhythmias. Signal processing advances could provide improved data quality impacting clinical translation. The aim of this study was to assess the fetus with known or suspected tachycardia using noninvasive abdominal fECG and correlate results with fetal echocardiography and postnatal ECG.

**METHODS:**

Prospective recruitment of pregnant participants with known or suspected fetal tachycardia in a tertiary fetal cardiology unit. Overnight fECG recording at home using the MonicaAN24 monitor was performed. Data processing using bespoke MATLAB scripts was undertaken to produce fetal heart rate and beat-to-beat rhythm strips. Comparison of fECG data with clinical data obtained using echocardiography and postnatal findings. Data are presented as median (interquartile range; range).

**RESULTS:**

Fifteen participants undertook 1 to 4 fECG recordings, giving a total of 23 recordings. Gestational age was 28.9 (23.9–34.3; 21–39.1) weeks. Duration of recording was 512 (380–609; 5–1259) minutes. Intermittent tachycardia was demonstrated on fetal heart rate graphs. Rhythm strips correctly identified short-ventriculoatrial and long-ventriculoatrial tachycardia, atrial flutter, and sinus rhythm with findings correlating with echocardiography. Postnatal ECG correlation was possible in 3.

**CONCLUSIONS:**

We have shown that rhythm strips of fECG signals can be extracted and correctly identify the electrical mechanism of arrhythmia in cases of fetal tachycardia. The potential to monitor fetal heart rate over a prolonged period is an advantage over current monitoring strategies for documentation of intermittent arrhythmias and gauging the response to medical therapy. These data will enable research to focus on improvement in signal quality, assessment of other arrhythmia subtypes, and real-time ambulatory monitoring of the fetal rhythm.

**WHAT IS KNOWN?**

- Accurate information on the electrical mechanism of fetal tachycardia can be obtained using fetal magnetocardiography.
- Application of the noninvasive abdominal fetal ECG has been limited previously due to background noise, leading to poor signal quality.

**WHAT THE STUDY ADDS**

- Advanced signal processing techniques can extract the beat-to-beat fetal ECG and enable correct identification of the electrical mechanism of fetal tachycardia.
- Ambulatory overnight monitoring captures data over a prolonged period, enabling the clinician to gain additional information on fetal heart rate, intermittent arrhythmia, and response to transplacental therapy.

The prevalence of fetal arrhythmia is reported as 1% to 2% of pregnancies, most commonly presenting as premature atrial contractions with a benign course.^1,2^ Fetal tachycardia, defined as a fetal heart rate (FHR) over 180 to 200 bpm, has the potential for serious and life-threatening consequences, including ventricular dysfunction, hydrops, and fetal demise.^3,4^ The most common tachycardias are supraventricular tachycardias (SVTs), including atrioventricular reentry tachycardia, atrial ectopic tachycardia, and permanent junctional reciprocating tachycardia in 66% to 90% and atrial flutter (AFl) in 10% to 30%.^5–8^

Following diagnosis, regular surveillance is required with consideration of maternal transplacental therapy. Current noninvasive diagnostic and monitoring strategies rely on intermittent Doppler auscultation of FHR and fetal echocardiography. While these techniques are accurate and well tolerated, they cannot provide direct electrophysiological information. Furthermore, none of these techniques are scalable or practical to be used in the home environment or remote settings.

To noninvasively assess the fetal electrical impulses and provide accurate information on arrhythmia mechanism, there are currently 2 options, limited to research settings. The first, fetal magnetocardiography, is the gold standard, including in some cases where echocardiography has not provided a full diagnosis.^9^ Use is limited to a few worldwide centers, but there is a potential for expansion in the modality secondary to the development of cost-effective optically pumped magnetometers.^10^

The second is noninvasive abdominal fetal ECG (fECG) with the ability to assess and monitor FHR and rhythm like a Holter monitor. Signal averaging is also possible to derive ECG time intervals such as the QT interval. Extracting the fECG from both the maternal signal and background noise has limited clinical applicability, but advances in signal processing techniques have enabled researchers to extract high-quality ECGs and interpret the derived traces and quantitative parameters.^11–14^ Our group has published on the normal fetal cardiac time intervals and heart rate variability patterns in pregnancies complicated by gestational diabetes utilizing the fECG.^15,16^

This study aimed to use fECG to assess the fetus with known or suspected fetal tachycardia. The derived results were correlated with the clinical findings obtained using echocardiography.

## Methods

We prospectively recruited and performed noninvasive fECG on participants over 20 weeks of gestational age who gave written informed consent with known or suspected fetal tachycardia. All participants had a fetal cardiology clinical consultation including echocardiography before fitting the fECG monitor.

Participants were recruited from the fetal cardiology department at Evelina London Children’s Hospital, London, United Kingdom. Ethical approval was obtained for the study REC15/WM/0017 alongside local institutional approval. Data were collected between December 21, 2021, and January 25, 2024. The data that support the findings of this study are available from the corresponding author upon reasonable request.

The fECG was recorded using the MonicaAN24 monitor (Monica Healthcare Limited, Nottingham, United Kingdom), fitted in the standardized manner by skin preparation and placement of 5 electrodes across the maternal abdomen.^17^ Inclusion criteria were singleton pregnancies with a known or suspected diagnosis of fetal tachycardia made by a fetal cardiologist. Exclusion criteria were multifetal pregnancies and women in active labor. The monitor was fitted in the clinic setting, worn overnight at home, and returned at the next clinical review.

Data were collected on participant demographics (recruitment gestational age, maternal medical and obstetric history, family history, and medications), fetal cardiology diagnosis, and postnatal outcome.

### Data Analysis

#### ECG Data Processing

The ECG data were preprocessed with the MonicaDKv1.9 software (Monica Healthcare Limited) by extraction of the raw fetal and maternal ECG data. The data were then imported into MATLAB R2023b (Mathworks, Inc, Natick, MA) where the abdominal ECG signals were modeled as the sum of maternal, fetal, and noisy contributions. A rough estimate of the fECG signal was obtained by subtracting the denoised maternal ECG signal from the linear combination of 2 (of 4) available channels. Using techniques similar to the maternal R-peak detection and denoising, the fECG is computed. This approach is iteratively applied to each of the linear combinations of the available channels (channel 1 and channel 2, channel 1 and channel 3, and so on). The best pair of channels is selected using the criterion of signal quality index, and the pair with the highest signal quality index is chosen as previously described.^18^

Each recording produced an overall FHR trace, and it was possible to interrogate each 1-minute segment of the recording such that maternal, fetal, and mixed maternal-fetal rhythm strips could be viewed on a beat-to-beat basis. Signal-averaged ECGs were not produced. Each case was assessed in the following way: overall assessment of the FHR trace, assessment of 1-minute segments of recording every 5 minutes for the whole recording, and assessment of any sudden change in FHR (defined as a 30-bpm change). Full analysis of a single trace using these methods could be performed within 24 hours of the monitor being returned. The data were analyzed for each case by S.C., and the results were further scrutinized by the referring clinician, W.R., E.R., and J.M.S.

Data were assessed for normality and are presented as median (interquartile range; range). Due to the small number of heterogeneous cases, no further statistical analyses were performed.

## Results

Fifteen participants were recruited, and all cases produced data suitable for analysis. Participants wore the monitor between 1× and 4× during the pregnancy, and the duration of recording was 512 (range, 5–1259) minutes. Twenty-three recordings were obtained. Case 9 obtained 5 minutes of recording due to a technical issue with the equipment. The remaining recordings were >242 minutes. Gestational age was 28.9 (23.9–34.3; 21.0–39.1) weeks. No case had fetal hydrops. Two cases had congenital heart disease, which was a ventricular septal defect. Summarized participant data are shown in Table 1.

**Table 1.**
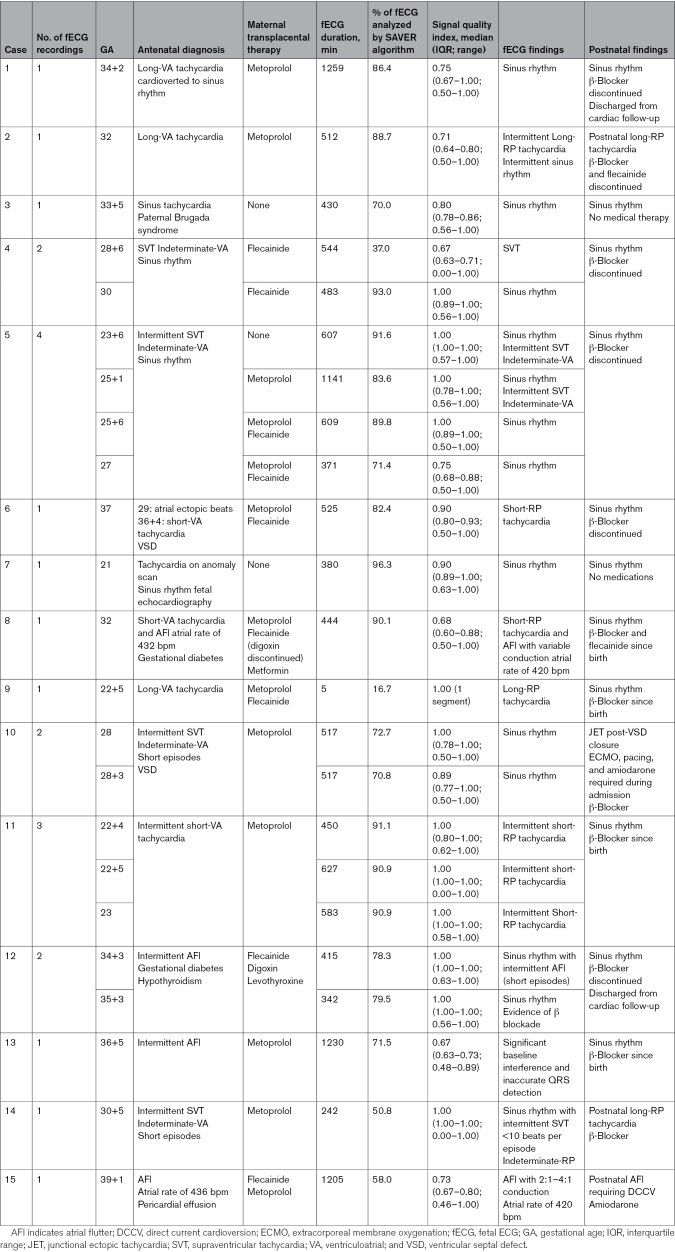
Summarized Data on Participants Including Background, Echocardiography Findings, fECG Findings, and Postnatal Outcome

Overall signal quality assessed using the signal quality index as detailed in the Methods section (reference range, 0–1) was 0.90 (0.78–0.96). The percentage of fECG recordings processed for analysis using the algorithm was 82.4% (70.8%–90.9%).

Interpretable FHR data were available in 22 of 23 (96%) recordings and interpretable fetal rhythm strip data in 22 of 23 (96%) recordings. For the fetal rhythm strips, 4 of 22 (18%) produced recordings where the QRS component only could be clearly defined. In the remaining cases, there was P- or P- and T- wave delineation. One case did not produce data that were interpretable due to noise throughout.

The fetal cardiology diagnosis was long-ventriculoatrial tachycardia in 3, short-ventriculoatrial tachycardia in 2, SVT with unspecified ventriculoatrial interval in 5, AFl in 4, and sinus tachycardia in 1.

### SVT With 1:1 Conduction

**Figure 1.**
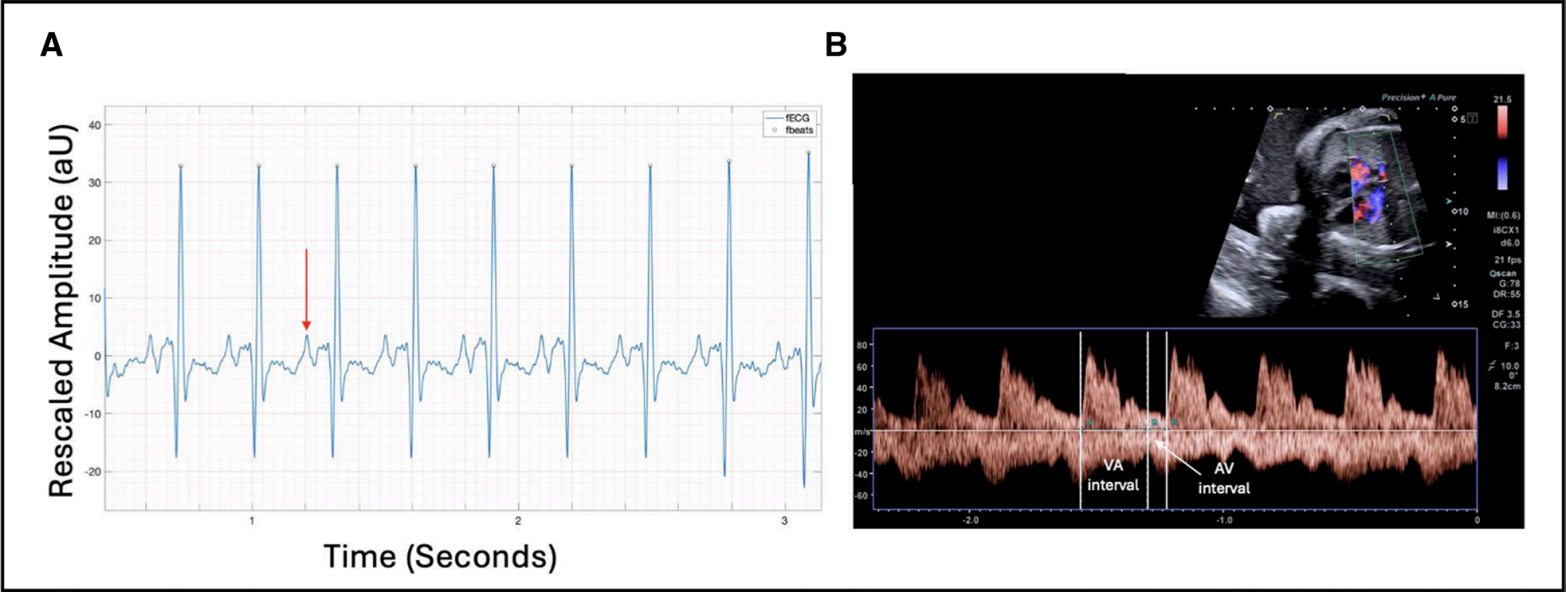
**Supraventricular tachycardia with long-ventriculoatrial (VA) interval (case 2). A**, Three-second fetal ECG (fECG) rhythm strip showing long-RP interval with P wave shown (red arrow). **B**, Corresponding Doppler trace of pulmonary vein and pulmonary artery trace with VA and AV interval marked. AU indicates arbitrary units.

Of the long-ventriculoatrial tachycardias, case 1 had been established on transplacental metoprolol and was in sinus rhythm on echocardiography at the time of recording. fECG demonstrated FHR of 134 (120–139) bpm throughout, with sinus rhythm on rhythm strip. Cases 2 and 9 had a maximum FHR of 200 bpm, and the long-RP signal was identified on the fetal rhythm strip (Figure 1; Figure S1).

**Figure 2.**
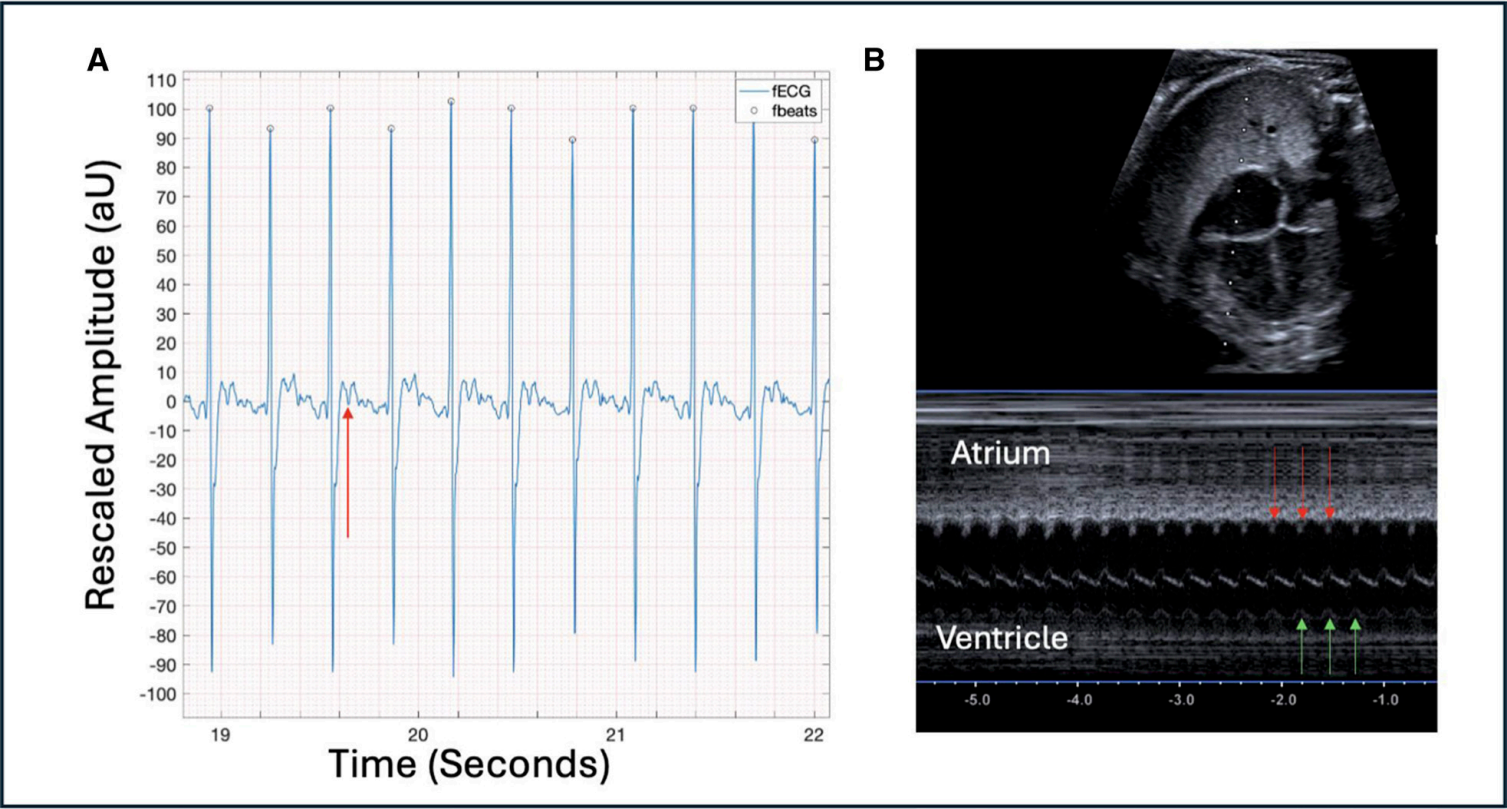
**Supraventricular tachycardia with short-ventriculoatrial interval (case 6). A**, Three-second beat-to-beat fetal ECG (fECG) rhythm strip showing short-RP interval with retrograde P wave shown (red arrow). **B**, M-mode of atria and ventricles showing atrial contractions (red arrows) and ventricular contractions (green arrows). AU indicates arbitrary units.

**Figure 3.**
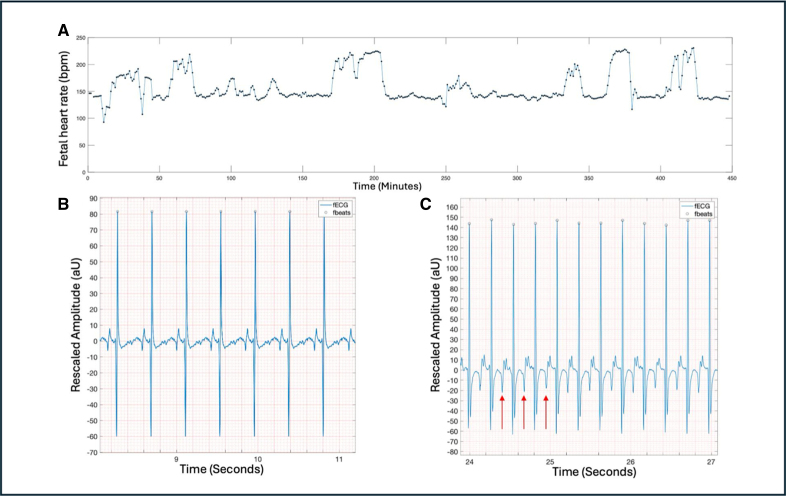
**Intermittent short-ventriculoatrial supraventricular tachycardia fetal ECG (fECG) findings (case 11). A**, Fetal heart rate trace demonstrating baseline fetal heart rate of 145 bpm with peaks to over 200 bpm. **B**, fECG panel from a 1-minute segment showing sinus rhythm. **C**, fECG panel from a 1-minute segment showing supraventricular tachycardia with retrograde P waves shown (red arrow). AU indicates arbitrary units.

Two cases had a diagnosis of short-ventriculoatrial tachycardia. Case 6 demonstrated tachycardia for most of the recording and a short-RP electrical signal on the fetal rhythm strip (Figure 2). Case 11 underwent 3 recordings. The FHR trace showed periods of sinus rhythm (145 bpm) and periods of tachycardia (235 bpm). Duration of time in tachycardia could be quantified by evaluation of FHR traces and individual 1-minute fetal rhythm strips evaluated to assess rhythm (Figure 3).

Five cases had 1:1SVT with an indeterminate ventriculoatrial interval. Case 4 had 2 fECG recordings, before and after establishing transplacental flecainide. The first recording showed tachycardia of 215 (185–219) bpm throughout and the second, normal FHR of 138 (131–178) bpm. Fetal rhythm strips from the first recording clearly delineated fetal QRS complexes corresponding to the FHR trace, but P waves were not discernible, and the underlying electrical mechanism could not be defined. The fetal rhythm strip from the second recording showed a sinus rhythm.

**Figure 4.**
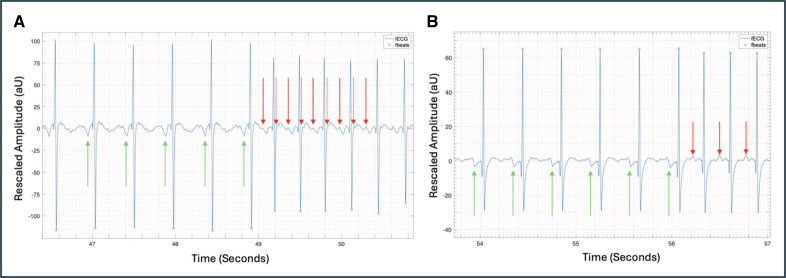
**Onset of fetal tachycardia. A**, Case 12, atrial flutter. Sinus beats are shown with green arrows; flutter waves are shown with red arrows. **B**, Case 14, 1:1 supraventricular tachycardia with indeterminate prenatal ventriculoatrial interval and postnatal long-RP tachycardia. Sinus beats are shown with green arrows. P waves during tachycardia are shown with red arrows. AU indicates arbitrary units.

The remaining 4 cases with indeterminate ventriculoatrial interval had short episodes of intermittent tachycardia on echocardiography (cases 5, 7, 10, and 14). Case 5 presented with short episodes of tachycardia lasting a few seconds. Four fECG recordings were performed. The FHR traces showed spikes from 140 to 200 bpm, and rhythm strips showed sinus rhythm, atrial ectopic beats, and intermittent tachycardia. Case 14 had intermittent short runs of SVT treated with transplacental metoprolol. The FHR trace showed a normal FHR throughout, but interrogation of fetal rhythm strips showed several episodes of short-lived tachycardia between 4 and 10 beats (Figure 4B). In case 7, possible SVT was seen during the anomaly scan and sinus rhythm during fetal cardiology assessment. Sinus rhythm was seen on the fECG recording with no tachycardia. Case 10 had 1 episode of very fast SVT seen during follow-up for VSD prenatally. Two recordings were performed, both of which showed sinus rhythm. No further SVT was seen in fetal life.

### Atrial Flutter

**Figure 5.**
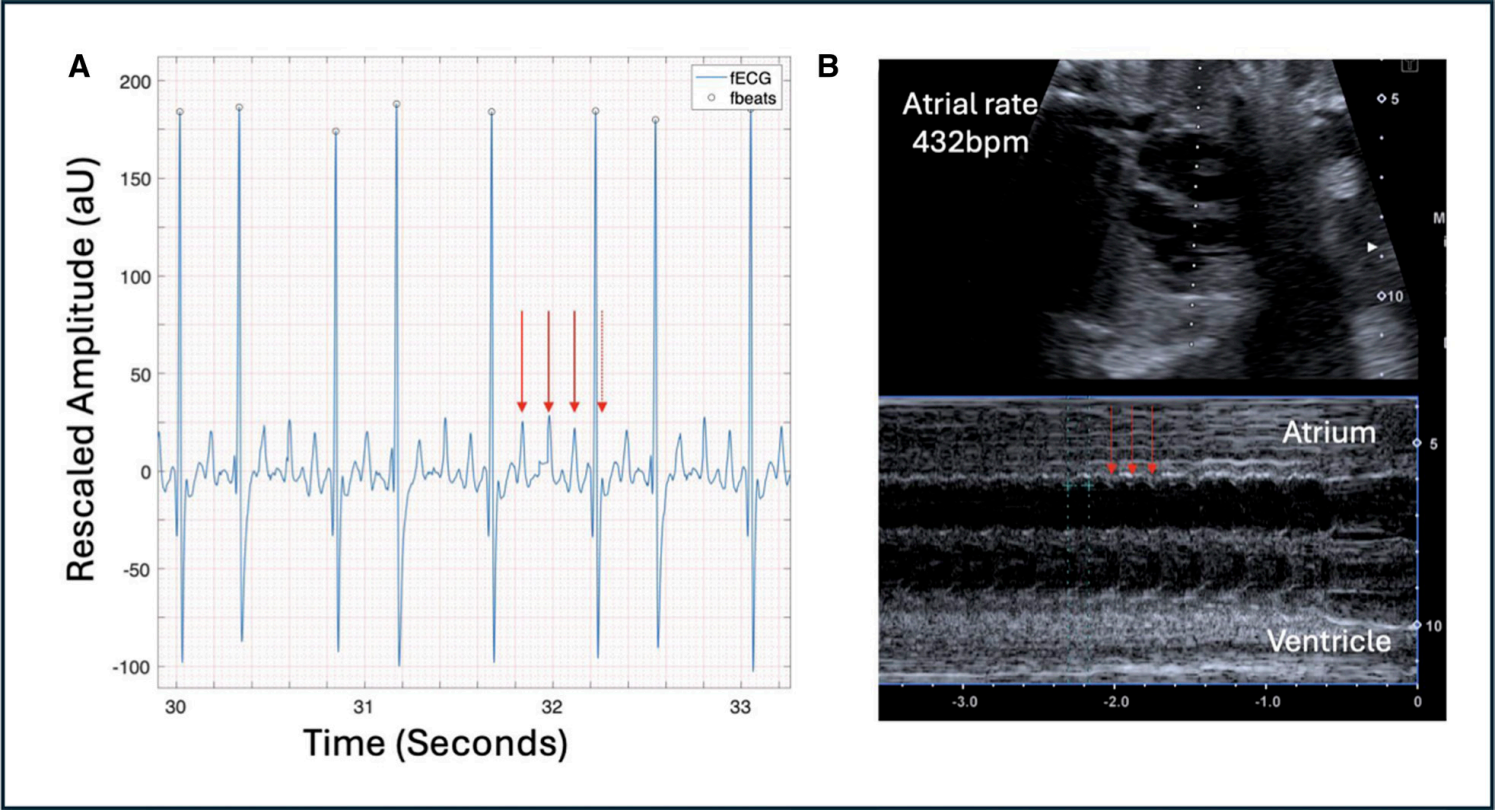
**Atrial flutter (case 8). A**, Three-second fetal ECG (fECG) rhythm strip showing atrial flutter with atrial rate of 420 bpm with variable conduction. P waves are shown with red arrows (final red arrow showing location of P wave embedded within QRS complex). **B**, M-mode of atria and ventricles showing atrial contractions (red arrows). Atrial rate, 432 bpm. AU indicates arbitrary units.

**Figure 6.**
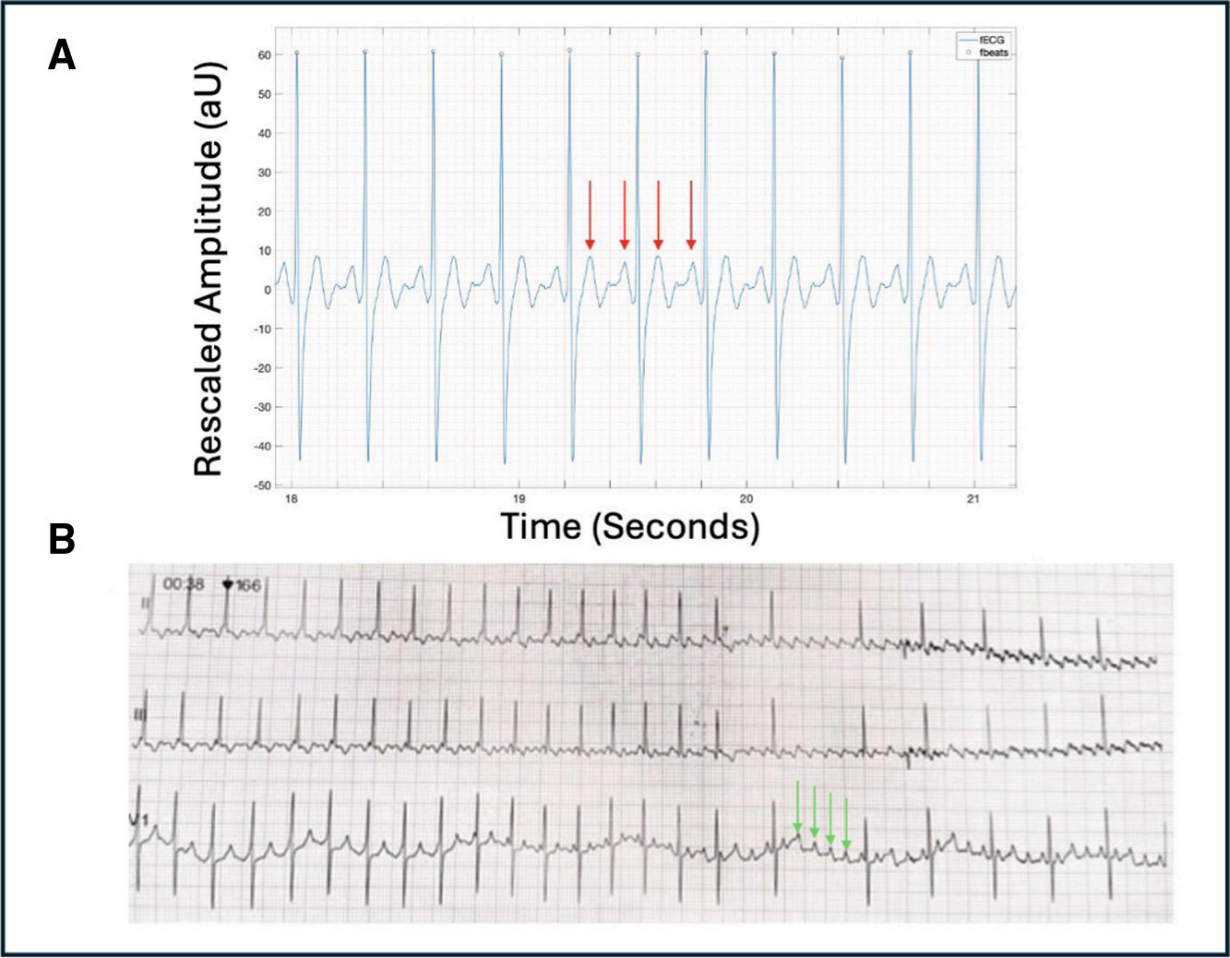
**Atrial flutter (case 15). A**, Three-second beat-to-beat fetal ECG (fECG) rhythm strip showing atrial flutter with 2:1 conduction and atrial rate of 420 bpm. P waves are shown (red arrow). **B**, Leads II, III, and V1 from postnatal ECG during adenosine administration unmasking underlying flutter waves (green arrows). AU indicates arbitrary units; and VA, ventriculoatrial.

Four cases had a diagnosis of AFl. Case 8 had a mixed picture of short-ventriculoatrial tachycardia and AFl with variable block. The FHR trace showed a ventricular rate of 158 (145–175) bpm. Rhythm strips showed AFl with variable block and short-RP tachycardia. Atrial rate on fetal rhythm strip (420 bpm) correlated with atrial rate on echocardiography (436 bpm; Figure 5). Case 12 had intermittent AFl and underwent 2 fECG recordings. The first FHR trace showed baseline spikes in FHR to 180 from 140 bpm. Fetal rhythm strips showed intermittent AFl with 2:1 conduction corresponding with echocardiography (Figure 4A). The second recording after establishing transplacental digoxin and flecainide showed sinus rhythm (FHR, 130–160 bpm) with no AFl on rhythm strips. Case 15 was seen as a follow-up for intermittent atrial ectopics where AFl was diagnosed with an atrial rate of 436 bpm. The fECG findings correlated with FHR of 180 to 200 bpm and AFl seen on fetal rhythm strips with variable and 2:1 to 4:1 ventricular conduction (Figure 6; Figure S2).

Case 13 underwent fECG recording at 36+5 weeks of gestational age. There was electrical noise throughout, which meant that QRS detection was not accurate and FHR could not be computed. The fetal rhythm strips were not interpretable.

### Sinus Rhythm

Case 3 had sinus tachycardia and a family history of Brugada syndrome. FHR was 138 (120–148) bpm with no excursions >180 bpm. Fetal rhythm strips showed sinus rhythm. Successive fetal cardiology review showed normal FHR and no ongoing concern.

Summarized data on fECG findings stratified according to type of rhythm are shown in Table 2.

**Table 2.**
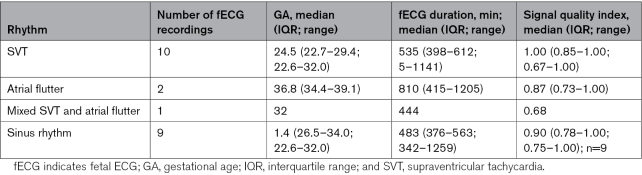
Summarized Data on fECG Findings Stratified According to Type of Rhythm (fECG Recording From Case 13 Excluded as Significant Baseline Interference and Inaccurate QRS Detection)

### Outcomes

All fetuses remain alive at follow-up. Postnatally, there was a recurrence of tachycardia in 4. Two had postnatal long-RP tachycardia (cases 2 and 14), 1 had postnatal AFl (case 15), and 1 had junctional ectopic tachycardia following VSD repair (case 10). Paired fetal and postnatal ECGs: case 15 (Figure 6), case 14 (Figure S3), and case 2 (Figure S4).

## Discussion

This study demonstrates our initial experience of the utility of fECG in the context of fetal tachycardia. Several types of tachycardia have been shown including short-RP and long-RP tachycardia, AFl, and intermittent tachycardia alongside sinus rhythm. It has also shown utility in the assessment of the electrical signal using prolonged overnight recordings where both FHR graphs and beat-to-beat fetal rhythm strips can be extracted. Response to transplacental therapy has been demonstrated with recordings captured before and after initiation of transplacental therapy. Findings correlated with the gold standard for clinical assessment of these fetuses using echocardiography and postnatal findings.

FHR parameters could be assessed in 22 of 23 (96%) of recordings with a median recording duration of 512 minutes. While we were not able to provide complete data for all recordings, the data obtained were significantly greater than would be gathered during a routine clinic assessment (30-minute to 1-hour scan time) and provided complementary information on FHR control. No other published studies have demonstrated this utility where there is fetal arrhythmia and FHR outside of the normal range. The MonicaAN24 monitor concurrently assesses maternal heart rate, and this functionality could be investigated, such as in cases where maternal transplacental therapy is used.

FHR assessment is commonly used to assess fetal well-being with the most common methods being via handheld Doppler and cardiotocography. These are useful for screening all pregnancies at antenatal appointments and during labor but have limitations. Handheld Doppler is subjective and provides a short monitoring duration. Cardiotocography requires hospital admission and can lead to inaccuracies in FHR measurement because it averages the FHR over 2 to 3.75 seconds rather than assessing the true beat-to-beat FHR. It will commonly underestimate very high FHRs >200 bpm, meaning that significant fetal tachycardia cannot accurately be monitored. The noninvasive abdominal fECG is portable and has been used in several studies to assess FHR including the safe passage study of over 4000 fetuses.^19^ It has, however, been less robustly tested in the assessment of the fetus with abnormal heart rate.^13^ In the postnatal population, Holter monitoring is the gold standard for noninvasive assessment of heart rate (over hours-days duration),^20^ and the noninvasive abdominal fECG provides the most similar modality available in fetal life.

Our results enabled us to pinpoint fetuses where there was short-lived intermittent tachycardia <1 minute (cases 5, 12, and 14) and those of longer duration of 20 to 30 minutes (case 11). These data provide direct clinical benefit because prolonged recordings enable quantification of the duration of time in tachycardia, which can be conveyed to the clinician directly and correlated with clinical findings. This is important because intermittent SVT has known associated adverse outcomes for the fetus including hydrops, increased risk of prenatal and postnatal death, and high risk of recurrence in the postnatal period.^21^ Because intermittent arrhythmia may not manifest during clinic assessment, all fetuses with nonimmune hydrops are recommended to undergo fetal cardiac assessment so that this important diagnosis is not missed, and portable prolonged monitoring would be of benefit to these patients.

Management of tachycardia within our unit comprises regular review, weekly or twice weekly, until sinus rhythm is achieved, plus potential requests to local service providers to perform intermittent Doppler auscultation. While regular assessment of fetal rhythm and well-being is performed, disadvantages remain as both echocardiography and intermittent Doppler involve short monitoring duration, and the cardiac center may not be local to the patient. The benefit of home intermittent Doppler auscultation in pregnancies where anti-Ro antibodies are present has been investigated. Researchers found that it provided good patient uptake, and of the 21 mothers who identified an abnormal FHR, medical attention was sought within 12 hours in 14 cases.^22^ While this option could be useful, it relies on intermittent subjective assessment by the patient. A monitor that objectively records FHR accurately would be preferable, but signal transmission would need to be organized to enable rapid clinician review and action. This is achievable in our wireless cloud-based systems of today.

In our series, there were fECGs performed before and after initiation of transplacental therapy where response to therapy could be gauged. Clinicians currently have the advantage of data related to drug therapeutic levels, such as flecainide, where there are published data on dose response,^23^ but complementary information on FHR and rhythm would give additional clinical insight. While the FHR graphs can provide information on elevated FHR, when there were short bursts of tachycardia of a few beats, it did not elevate the 1-minute average FHR; therefore, strategies should be used when developing analysis scripts for the fECG so that these cases are not missed through the use of the beat-to-beat data and fetal rhythm strips.

The fECG device used in this study (MonicaAN24) was provided for home monitoring for the participants. Recordings were performed overnight when there is less body movement, optimizing high-quality output and giving hours of data for analysis. This method proved to be successful with no failed recordings. The monitor was returned at the next clinical visit, and feedback was provided to participants. Turnaround of results was possible in 24 hours. This method is scalable due to the size and portability of the device and its relatively cheap cost compared with fetal magnetocardiography. In addition, it has the potential for dissemination to remote and low-income settings due to outputs of the monitors being transmissible electronically. This has significant implications, as it offers the potential to not only monitor pregnancies with known arrhythmia but also as a tool to access high-risk women who may not be able to access fetal cardiology services.

### Use of Artificial Intelligence

Our results were analyzed manually. Due to the amount of data collected, it was cumbersome to analyze every minute of each recording. Given the data modality, it would be appropriate to train artificial intelligence software to process and analyze this data set, which may provide additional insight. Computer scientists have investigated the potential for deep learning on the fECG.^24^ In addition, artificial intelligence packages exist for the detection of abnormalities of rhythm. One such application lies in the identification of atrial fibrillation within the adult population. Screening strategies for atrial fibrillation remain flawed using single 12-lead ECGs because atrial fibrillation may be paroxysmal and asymptomatic.^25,26^ Artificial intelligence packages have been developed, which can predict atrial fibrillation based on a 12-lead ECG in sinus rhythm with an area under the receiver operator curve of 0.87.^27^

Numerous studies have assessed the fECG, most commonly using fetal magnetocardiography. Fetal MCG is the gold standard for assessment of the fetal rhythm and has been shown to reliably assess the fetal cardiac time intervals and rhythm disturbances^9^; however, use has not been broadly adopted secondary to cost implications and inability to provide ambulatory recordings. Developments in the modality are improving accessibility as technology cost is decreasing secondary to the release of optically pumped magnetometer sensors and person-sized magnetic shields offering an alternate approach to gaining fECG signals.^10,28^

### Study Benefits and Limitations

This study has shown the benefits of noninvasive abdominal fECG in fetal tachycardia using methods, which have potential for translation into clinical practice. It has given information from prolonged recordings, which are of particular use to gauge response to therapy and assess intermittent arrhythmias.

While the recordings were conducted overnight, they were still subject to interference, leading to signal loss and noise impacting on reliability of all data despite thresholds put in place by the algorithm to discount low-quality signals. Further work is needed to improve processing algorithms so that areas of high quality are represented. Larger participant numbers with fetal arrhythmia would help improve processing techniques and consideration of clinical translation. Collaboration with other groups and sharing of processing code could help tailor the analysis scripts so that they can be improved for clinical utility.

### Conclusions

Noninvasive abdominal fECG can be collected and interpreted successfully in several different tachycardia types, providing useful information on both FHR and arrhythmia mechanism. Additional potential uses of the fECG data include response to transplacental therapy and interpretation of maternal ECG data. These results could provide a successful framework for the implementation of a system that could collect fECG remotely. Paired with the intelligent algorithm, this demonstrates effort toward the investigation of pathological fetal rhythms. Future directions could improve the algorithm, assess other arrhythmia subtypes, and investigate validation toward an automated system with analyzed results presented to the clinician.

## ARTICLE INFORMATION

### Sources of Funding

The work of S. Chivers was supported by Diabetes UK, Sir George Alberti Fellowship (grant 21/0006277).

### Disclosures

Dr Hayes-Gill was the Research Director at Monica Healthcare Ltd (designer and manufacturer of the AN24) from May 2005 to July 2018. Since this date, Dr Hayes-Gill has not had any formal engagement with Monica Healthcare Ltd. This manuscript was planned after July 2018. The other authors report no conflicts.

### Supplemental Material

Figures S1–S4

## Supplementary Material
