# Supplementary material for: Clinical Role of the Noninvasive Abdominal Fetal ECG in the Detection and Monitoring of Fetal Tachycardia
Source: Circ Arrhythm Electrophysiol. 2025 Sep 11;18(9):e013556. doi: 10.1161/CIRCEP.124.013556 (PMC12442779; doi:10.1161/CIRCEP.124.013556)

SUPPLEMENTAL MATERIAL

Supplemental Figure 1: Supraventricular tachycardia with long-VA interval (Case 9). Panel A - three second beat to beat fetal ECG rhythm strip showing long RP interval with P wave shown (red arrow). Panel B - Corresponding Doppler trace of pulmonary vein and pulmonary artery trace with VA and AV interval marked. *(aU - arbitrary units)*

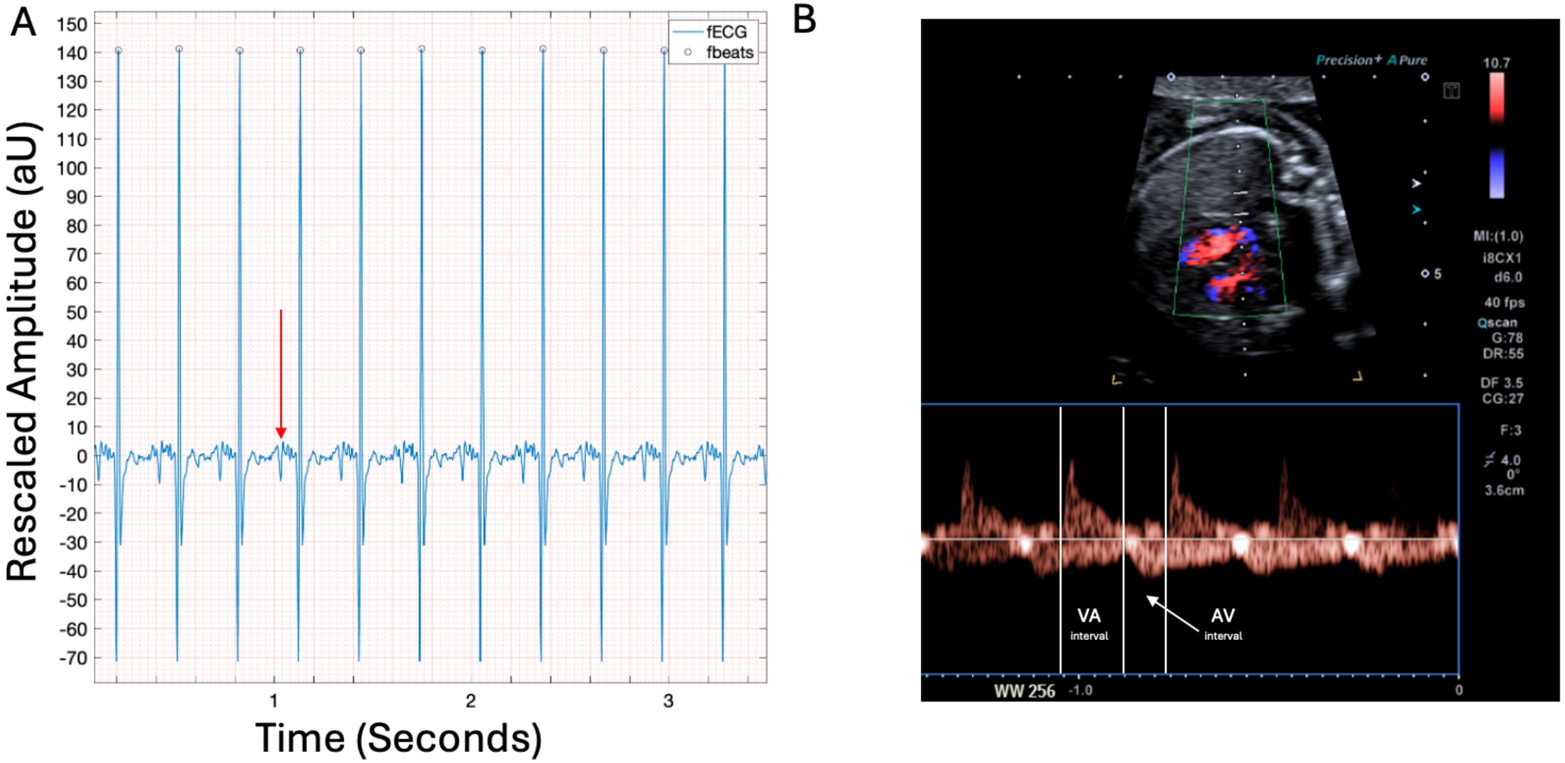

Supplemental Figure 2. Atrial flutter (Case 15). Panel A - three second beat to beat fetal ECG rhythm strip showing atrial flutter with 2:1 conduction and atrial rate 420bpm. P waves shown (red arrow). Panel B - M-mode of atrial and ventricles showing atrial contractions (red arrows). Atrial rate 436 bpm.
(au - arbitrary units)

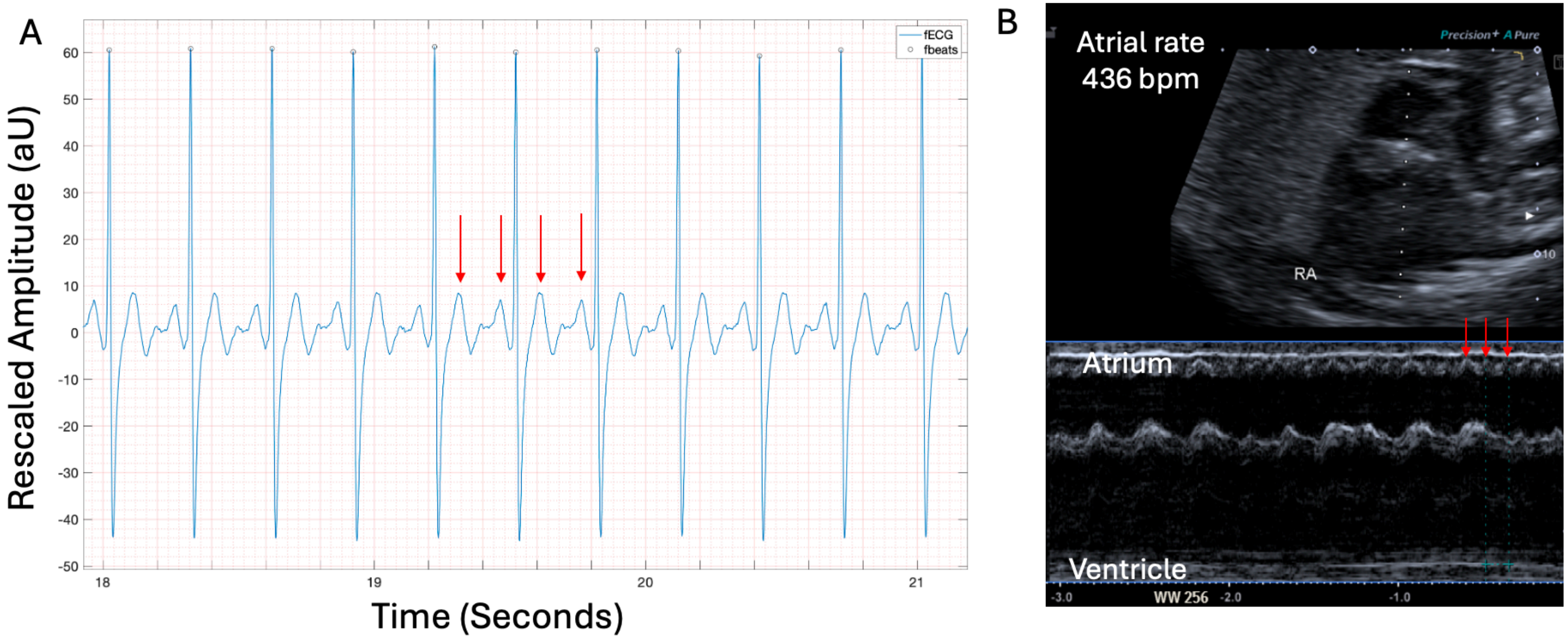

Supplemental Figure 3. 1:1SVT (Case 14) with intermittent episodes of long-RP tachycardia. Panel A - three second beat to beat fetal ECG rhythm strip showing two sinus beats prior to an episode of intermittent tachycardia with P-waves shown (red arrow). Panel B - Leads I, II, III, aVR, aVL and aVF from postnatal 12-lead ECG showing P-waves during tachycardia (green arrows). *(aU - arbitrary units)*

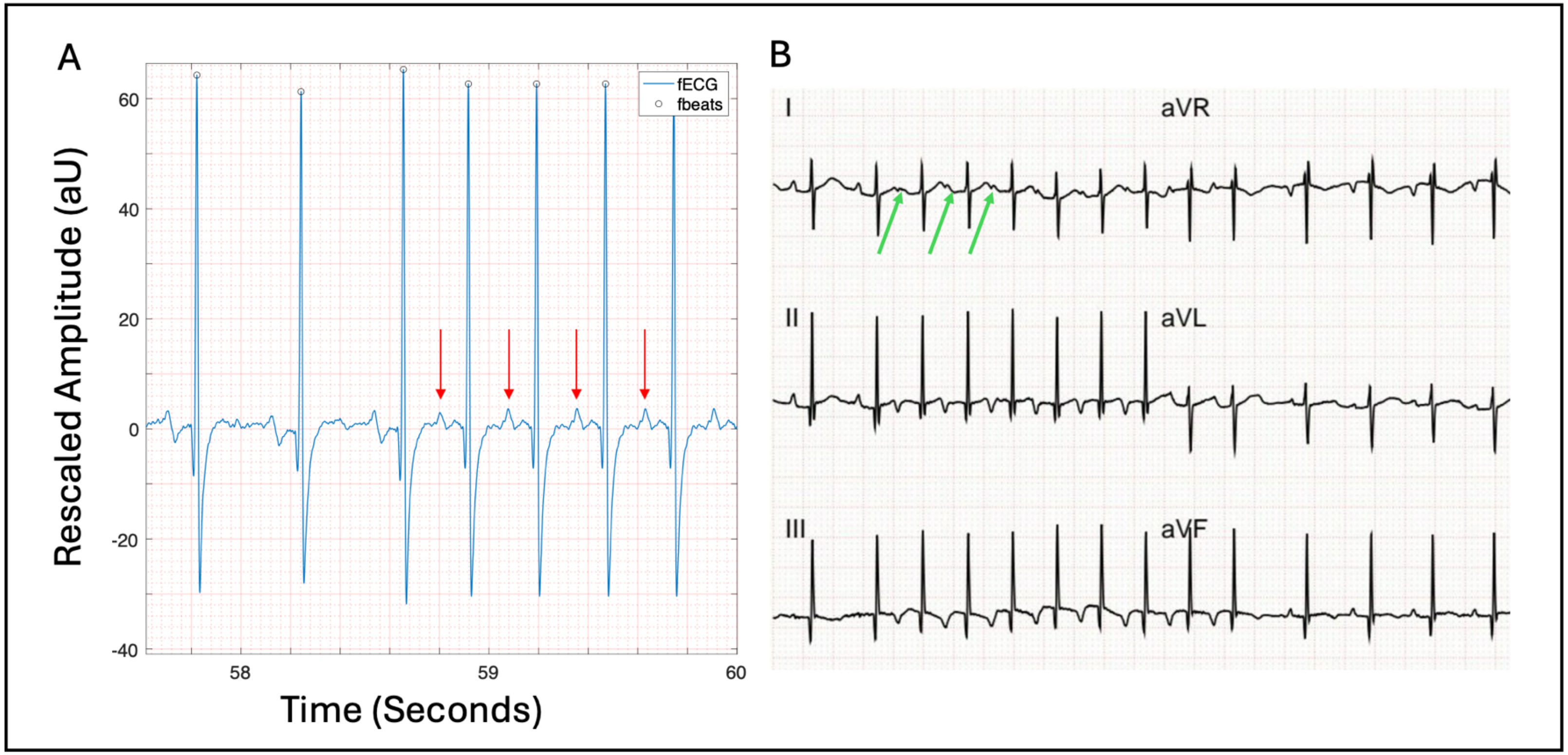

Supplemental Figure 4. Supraventricular tachycardia with long-VA interval (Case 2). Panel A - three second fetal ECG rhythm strip showing long-RP interval with P-wave shown (red arrow). Panel B - Leads V1-V4 from postnatal 12-lead ECG during tachycardia with P-waves shown (blue arrows). *(aU - arbitrary units)*

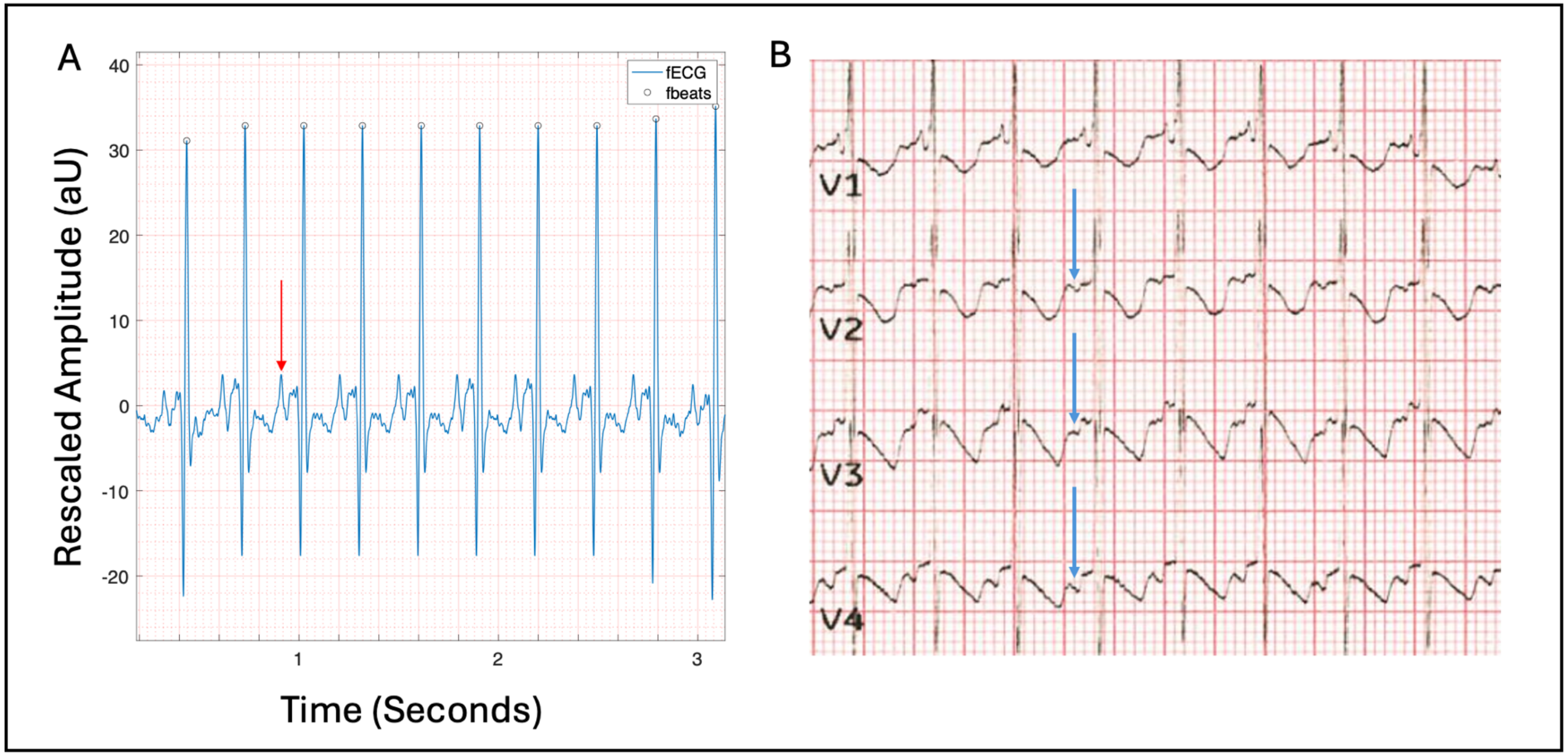

Supplement: Supplementary file 1 [file hae-18-e013556-s001.pdf]
